# RNA methyltransferase NSun2 deficiency promotes neurodegeneration through epitranscriptomic regulation of tau phosphorylation

**DOI:** 10.1007/s00401-022-02511-7

**Published:** 2022-11-10

**Authors:** Yoon A. Kim, Tohid Siddiqui, Jennifer Blaze, Mehmet Ilyas Cosacak, Tristan Winters, Atul Kumar, Ellen Tein, Andrew A. Sproul, Andrew F. Teich, Francesca Bartolini, Schahram Akbarian, Caghan Kizil, Gunnar Hargus, Ismael Santa-Maria

**Affiliations:** 1 Taub Institute for Research on Alzheimer’s Disease and the Aging Brain, Columbia University, New York, USA; 2 Department of Pathology and Cell Biology, Columbia University, New York, USA; 3 German Center for Neurodegenerative Diseases (DZNE), Helmholtz Association, Dresden, Germany; 4 Friedman Brain Institute, Icahn School of Medicine at Mount Sinai, New York, USA; 5 Department of Psychiatry, Icahn School of Medicine at Mount Sinai, New York, USA; 6 Department of Neurology, College of Physicians and Surgeons, Columbia University and the New York Presbyterian Hospital, New York, USA; 7 Present Address: Facultad de Ciencias Experimentales, Universidad Francisco de Vitoria, Edificio E, Pozuelo de Alarcón, Madrid, 28223 Spain

**Keywords:** Alzheimer’s disease, NSun2, Neurodegeneration, Tau phosphorylation, MicroRNA, Methylation

## Abstract

Epitranscriptomic regulation adds a layer of post-transcriptional control to brain function during development and adulthood. The identification of RNA-modifying enzymes has opened the possibility of investigating the role epitranscriptomic changes play in the disease process. NOP2/Sun RNA methyltransferase 2 (NSun2) is one of the few known brain-enriched methyltransferases able to methylate mammalian non-coding RNAs. NSun2 loss of function due to autosomal-recessive mutations has been associated with neurological abnormalities in humans. Here, we show NSun2 is expressed in adult human neurons in the hippocampal formation and prefrontal cortex. Strikingly, we unravel decreased NSun2 protein expression and an increased ratio of pTau/NSun2 in the brains of patients with Alzheimer’s disease (AD) as demonstrated by Western blotting and immunostaining, respectively. In a well-established *Drosophila* melanogaster model of tau-induced toxicity, reduction of NSun2 exacerbated tau toxicity, while overexpression of NSun2 partially abrogated the toxic effects. Conditional ablation of NSun2 in the mouse brain promoted a decrease in the miR-125b m6A levels and tau hyperphosphorylation. Utilizing human induced pluripotent stem cell (iPSC)-derived neuronal cultures, we confirmed NSun2 deficiency results in tau hyperphosphorylation. We also found that neuronal NSun2 levels decrease in response to amyloid-beta oligomers (AβO). Notably, AβO-induced tau phosphorylation and cell toxicity in human neurons could be rescued by overexpression of NSun2. Altogether, these results indicate that neuronal NSun2 deficiency promotes dysregulation of miR-125b and tau phosphorylation in AD and highlights a novel avenue for therapeutic targeting.

**Supplementary Information:**

The online version contains supplementary material available at 10.1007/s00401-022-02511-7.

## Introduction

MicroRNAs (miRNAs), a class of non-coding small RNAs, are part of a vital regulatory mechanism that prevents the deposition of tau protein [63, 75]. Several studies have reported miR-125b is significantly upregulated in the frontal cortex and hippocampus in Alzheimer’s disease (AD) brains [6, 20, 58, 78]. MiR-125b upregulation has been positively correlated with gray matter neurofibrillary tangles in post mortem AD patients [86]. Mechanistically, observed miR-125b upregulation induces tau hyperphosphorylation and cell death by targeting DUSP6 and PPP1CA phosphatases and activating cyclin-dependent kinase 5 (CDK5) and p44/42-MAPK signaling in neurons [6, 59]. Several miRNAs have been shown to regulate tau proteostasis by modulating tau synthesis or post-translational modifications on tau, such as phosphorylation [72]. However, mechanisms governing how miRNAs are regulated in the brain or how they are dysregulated during the disease process are not well understood [7, 31, 45, 47, 48, 68, 69].

miRNAs can be regulated at the transcriptional or post-transcriptional level [31, 55]. One of the most frequent post-transcriptional modifications of RNA is methylation [14, 25, 79, 95]. *N*^6^-Methyladenosine (m^6^A) and 5-methylcytosine (m^5^C) marks are prevalent post-transcriptional modifications of RNAs that regulate biogenesis, stability, accuracy of translation initiation and function of RNAs in a highly “dynamic” and “reversible” fashion. Recently, miRNAs have been found to be targets of m^6^A methylation [4, 46, 93]. However, the specific methyltransferase able to install m^6^A marks in miRNAs in the brain has yet to be described.

NSun2 is one of the methyltransferases known to facilitate methylation of non-coding RNAs [10, 39, 83, 93], whose loss of function due to autosomal-recessive mutations can cause disorders that are associated with intellectual disability in humans [1]. Recent studies have elucidated the first association between m^5^C regulation during cellular stress responses and other non-canonical functions of tRNAs in neurodevelopment and in human diseases [10]. These studies have shown that loss of NSun2 induces the accumulation of 5′ tRNA fragments that activate stress response pathways leading to reduced rates of protein translation, decreased cell size, decreased synaptogenesis, and increased cell death during mouse embryogenesis.

Interestingly, NSun2 seems to have a broader substrate specificity with the ability to facilitate installing of m^5^C or m^6^A depending on the type of RNA and the interacting partners of this methyltransferase [16, 29, 39]. Regarding miRNA methylation, it has been shown that NSun2 represses the levels and function of miR-125b via m^6^A methylation that inhibits the processing of miR-125b leading to overall reduction in the levels of the mature miR-125b [50, 90, 93]. In addition, NSun2-mediated m^6^A modification of miR-125b can attenuate the recruitment of the RNA-induced silencing complex affecting miR-125b induced silencing of RNA targets [93].

Based on the previously reported putative neuroprotective role of NSun2, its ability to regulate miR-125b and accumulating evidence supporting the role of RNA modifications in the physiology and pathology of the nervous system [10, 19, 24], we decided to investigate the status of NSun2 and its potential role in AD pathogenesis.

## Materials and methods

### Human brain tissue samples

Autopsy brain samples were obtained from the New York Brain Bank at Columbia University Medical Center. The demographics and postmortem neuropathological findings of human cases identified in the Columbia University Alzheimer’s Disease Research Center Neuropathology Core and used in this study are listed in Table 1 and in more detail in Supplementary Table 1 (online resource). These specimens were obtained by consent at autopsy and have been de-identified and are IRB exempt to protect the identity of each patient. Patients with no or limited age-related tau pathology and without amyloid pathology and without dementia were assigned to the control group for comparisons with AD patients, primary age-related tauopathy (PART) cases with dementia and progressive supranuclear palsy (PSP) patients (Table 1; Supplementary Table 1, online resource). Formalin-fixed paraffin-embedded (FFPE) specimens were sectioned by the Histology Service at Columbia University Medical Center. Immunohistochemistry was performed on 6 μm paraffin-embedded sections as previously described [76] using various antisera (see below). Images were captured using an Olympus BX53 microscope with an Olympus camera DP-72 (Olympus Lifescience).

**Table 1:** Case demographics

Classification	Number of subjects	Gender (male/female)	Age at autopsy (mean, SD and range; in years)	Clinical diagnosis of dementia	Braak stage	CERAD plaque score	PMI (hours)
AD	24	11/13	87.3 ± 7.4 (71–102)	24/24	IV–VI	B–C	2.4–22.3
PART	31	11/20	84.7 ± 10.5 (56–103)	10/31 (1 × MCI)	II–IV	0–0	1.1–26.9
PSP	10	9/1	68.4 ± 5.3 (55–75)	0/10	NA	0–0	0.6–6.9
Healthy individuals	7	5/2	78.1 ± 11.3 (62–93)	0/7	0	0–0	NA–33.6

*AD* Alzheimer's disease, *PART* primary age-related tauopathy, *PSP* progressive supranuclear palsy, *MCI* mild cognitive impairment, *PMI* postmortem interval, *SD* standard deviation

### Mice

All animal studies were performed according to protocols examined and approved by the Animal Use and Care Committee of Columbia University and Icahn School of Medicine at Mount Sinai. C57BL/6N-Nsun2^tm1c(EUCOMM)Wtsi^/WtsiOulu mutant mouse sperm was obtained from the Wellcome Trust Sanger Institute (Cambridgeshire, UK). Mice with the tm1c allele exhibit a phenotypically wild-type state although loxP sites flank exon 6 of Nsun2. Cre-recombinase excises this exon and produces a frameshift, resulting in early termination of NSun2 translation. In vitro fertilization was performed with C57BL/6 N-Nsun2^tm1c(EUCOMM)Wtsi^/WtsiOulu mutant mouse sperm and wildtype (control) C57BL/6 N females. Mice were then bred further to obtain *Nsun2*^*2lox/2lox*^ mice (2 copies of tm1c mutant allele). *Nsun2*^*2lox/2lox*^ mice were crossed with a *CamK-Cre*^+^ line [3, 18, 27, 80] to produce *CamK-Cre*^+^*, Nsun2*^*2lox/2lox*^ (NSun2 KO) mice for knockout of Nsun2 in excitatory forebrain neurons.

### Immunohistochemistry on mouse brain tissue

Brain embedded sections from 11-month-old NSun2 KO and controls (7 microns thick) were deparaffinized in Histo-Clear II (National Diagnostics, GA) and processed for immunohistochemistry using anti-mouse and rabbit tau antibodies (see below) according to the manufacturer’s protocol for mouse brain sections (MOM kit; Vector Labs, Cat # PK-2200). A 30 min incubation with 3% H2O2/10% methanol/0.25% Triton X-100 was used to block endogenous peroxidase activity. 3,3′-Diaminobenzidine was used as a peroxidase substrate (Vector DAB Substrate Kit for Peroxidase, Cat # SK-4100). Tissue sections were counterstained with hematoxylin (Vector Labs, Cat # H-3404) and mounted using VectaMount (Vector Labs, Cat # H-5000). Images were captured using an Olympus BX53 microscope with an Olympus camera DP-72 (Olympus Lifescience).

### Automated image quantification and statistical analyses

Human brain sections from hippocampus and frontal cortex of 9 controls and 8 AD patients were stained for NSun2 (see below). 178 random bright field images (90 control, 88 AD) were acquired with identical image acquisition parameters. Another set of human brain sections from hippocampus and frontal cortex of 5 controls and 8 AD patients were fluorescently immunostained for NSun2 and pTau. 231 random fluorescent images (90 control and 141 AD) were acquired with identical acquisition parameters. All images were analyzed using Arivis vision4D-based automated quantification (script available upon request). The analysis pipelines were designed to measure the total expression of NSun2 and pTau in individual cells. For bright field images, 4512 control cells and 3848 AD cells were analyzed for NSun2 expression levels. For fluorescent images, 1802 control cells and 3617 AD cells were analyzed for expression levels of NSun2 and pTau and their ratios.

Two pipelines were designed to perform quantifications on bright field images. The first pipeline quantified the total number of cells and masked the intensity of NSun2 expression in the nucleus. The second pipeline was used to measure the total sum intensity of NSun2 in the defined regions. For fluorescent image quantifications, Arivis vision4D machine learning tools were used (script available upon request). 5 random images were selected to train the Arivis AI. Trained data were used to quantify every fluorescent image and the total sum intensities of NSun2 and pTau was measured in the verified cells.

All analyses were performed in a blind manner. Samples were de-identified by one researcher and labeled randomly, imaging and automated quantification by performed by another researcher before unblinding and the raw data are shared with three other researchers, and finally data analyses and statistical measurements were performed by the third researcher after unblinding. Demographics were revealed after the quantifications of individual samples.

Summary statistics were prepared by non-parametric *t* test with Kolmogorov–Smirnov to compare cumulative distributions with 95% confidence interval for every variable (NSun2 intensity, pTau intensity and pTau/NSun2 ratios). One-sample *t* and Wilcoxon signed rank tests were used to confirm the significance for summary statistics. **p < 0.0021; ***p < 0.0002, ****p < 0.0001.

### *Drosophila* stocks

All *Drosophila* stocks were maintained on standard food (Bloomington recipe, Archon Scientific) in incubators at constant 70% relative humidity and 25 °C on a 12-h/12-h light/dark cycle. *Drosophila* dNSun2 line was generated and kindly shared with us by Dr. Stephan J. Sigrist [1]. EGFP control line (Stock #5430), NSun2-RNAi line (Stock #62,495) and GFP-RNAi control line (Stock #42,555) were obtained from Bloomington *Drosophila* Stock Center. For tau overexpression, we used the *Drosophila* line previously generated by us [75] with the full-length human tau coding sequence (2N4R). The GMR-GAL4 driver was used for expression of transgenes in the eye. For amyloid-beta overexpression, we used the *Drosophila* line previously generated by Dr. Fernandez-Funez [15] who kindly shared the animals with us. The GMR-GAL4 driver was used for expression of transgenes in the eye.

### Drosophila rough eye phenotype assessment

For light microscopy imaging of the *Drosophila* eyes, 7-day-old adult flies were collected, and eye images were recorded, in a blinded fashion, by three independent observers as described by others with slight modifications [73, 75]. Briefly, fly eye images of specified genotypes were acquired under a dissecting microscope by one researcher, coded and given to another two researchers for blind quantitative assessment. To accurately quantify eye degeneration, we used an Olympus Stereoscope SZX16 microscope in combination with the Qcapture Pro7 Imaging software. The extent of eye area undergoing degeneration was analyzed using the image processing and analysis program Image J (Image J, NIH) following previously described methods [41, 66]

### Cell culture

HEK 293 T cell line was used for optimal lentivirus production. HEK 293 T cells obtained from the American Type Culture Collection were grown in Dulbecco’s modified Eagle’s medium (ThermoFisher) supplemented with 10% fetal bovine serum, 2 mM glutamine, 100 units/ml penicillin, and 100 μg/ml streptomycin in a humidified atmosphere of 5% CO_2_ and 95% air at 37 °C.

Human induced pluripotent stem cells (iPSCs; IMR90, cl.4 backbone, WiCell) [17, 35, 92] were maintained feeder‐free in StemFlex media (ThermoFisher) and Cultrex substrate (Bio-techne). Transdifferentiation of human iPSCs into cortical-like pyramidal neurons was performed following established protocols [51]. Alternatively, neural progenitor cells were generated from iPSC controls, cultured and differentiated into neurons following established protocols [26].

Primary hippocampal neuronal cultures were prepared following previously established methods with mild modifications [53, 56]. Briefly, hippocampi were dissected from E18 rats, and neurons plated on 100 μg/mL poly-D-lysine-coated 12-well-plates at the density of 1.5 × 105 cells/well for biochemistry assays, or 6 × 104 cells/coverslip on 18 mm coverslips for immunofluorescence. Primary neurons were maintained in Neurobasal medium (ThermoFisher) with the B-27 supplement (ThermoFisher) and 0.5 mM glutamine (ThermoFisher) at 37 °C, and 1/3 of the medium was changed every 3–4 days up to 3 weeks in culture.

### Lentiviral shRNA preparation

The human pull of shRNA NSun2 and scramble shRNA control were obtained from Applied Biological Materials Inc. To prepare lentivirus for in vitro experiments, the second-generation packaging system (which generates replication-deficient lentivirus) was used for all experiments [99]. Packaging vectors such as psPAX2 and pMD2.G were obtained from Addgene. Briefly, lentiviral constructs for shRNA or scramble control were co-transfected with the packaging vectors into HEK293T cells using CalFectin (SignaGgen). Supernatants containing virus were collected 48 h after transfection. After centrifugation at 1000 rpm for 10 min, the supernatants were passed through a 0.45 μm PDVF filter unit (Nalgene). The viruses were concentrated 20–30 × by centrifugation in an Amicon Ultra centrifugal filter (100 K) (Millipore) following the manufacturer's instructions. Lentiviral stock titration was carried out using the Global UltraRapid Lentiviral Titer Kit (System Biosciences). Viruses were aliquoted and stored at − 80 °C.

### Site-directed mutagenesis

Site-directed mutagenesis of lentiviral plasmid enconding miR-125b was performed using the Q5 Site Directed Mutagenesis kit (Cat# E0554S) from New England Biolabs following the manufacture protocol using the required primers: Q5SDM_miR-125b_m6A motif_F CCTGAGACCCtggcTTGTGATAGTG and Q5SDM_miR-125b_m6A motif_R GAGTCGCTCACTGTCAAC. Substitution was confirmed by Sanger sequencing analysis.

### Amyloid-β oligomers preparation

Oligomer-enriched preparations of Aβ were obtained according to previously published methods [82]. Briefly, lyophilized Aβ_42_ peptide (rPeptide) was equilibrated to room temperature for 30 min to avoid condensation upon opening the vial. The lyophilized peptide was resuspended in hexafluoro-2-propanol (HFIP; Sigma-Aldrich) to a concentration of 1 mM to allow monomerization for another 2 h at room temperature and then aliquoted into low protein-binding Eppendorf tubes. HFIP was removed by speed vacuum, and the monomers were stored in − 80 °C. To prepare oligomer-enriched preparations, the aliquots were resuspended in anhydrous DMSO to make a 5 mM solution followed by 10 min of sonication. The resuspended peptide was diluted to 100 µM in ice-cold Ham’s F-12 medium, immediately vortexed for 30 s, and then incubated at 4 °C for 24 h before use. Total Aβ concentration was measured by bicinchoninic acid protein assay (BCA) after oligomerization and indicated concentration of oligomeric Aβ was used (see Supplementary Fig. 9, online resource). For control experiments, vehicle treatment corresponding to the same volume of DMSO and F12 media used for AβO treatment was processed as for Aβ_42_ oligomerization.

### Tissue and cell lysates preparation for Western blotting

Samples from human brain were stored at − 80 °C and ground with a mortar in a frozen environment with liquid nitrogen to prevent thawing of the samples resulting in tissue powder. Mouse brains were quickly dissected on an ice-cold plate and the different brain structures stored at − 80 °C. Protein extracts were prepared by homogenizing brain structures in ice-cold extraction buffer [250 mM sucrose, 20 mM Tris–HCl (pH 7.4), 1 mM ethylenediaminetetraacetic acid (EDTA), 1 mM ethylene glycol tetraacetic acid (EGTA)] containing cocktail of protease and phosphatase inhibitors (Halt Protease & Phosphatase Inhibitor Single-Use Cocktail; ThermoFisher) using 20 strokes with a Teflon-coated pestle. Homogenates were centrifuged at 3000 rpm for 5 min at 4 °C. The resulting supernatant was collected, and protein content determined by BCA (Pierce, Rockford, IL, USA).

To prepare cell lysates for Western blotting neurons were collected in 1X Laemmli sample buffer (150 ml/well on 24 well plates; Bio-Rad Laboratories). Cells were lysed and boiled for 5 min. A volume of 15 ml was subsequently used for electrophoresis.

### Antibodies used in this study

Rabbit anti-NSun2 (Cat# 20854-1-AP; IHC 1:500, IF 1:100 and WB 1:1000) and mouse anti-NSun2 (Cat# 66580-1-Ig; IF 1:500) antibodies were purchased from Proteintech. Rabbit anti-NSun2 (Cat# 702036; IF 1:250 and WB 1:500) was purchased from ThermoFisher Scientific. Mouse anti-m6A (Cat# 202 111; IP see amount used per ug of RNA) antibody was purchased from Synaptic system. Mouse anti-β-actin (Cat# A1978; WB 1:2000) was purchased from Sigma-Aldrich. Rabbit anti-β-III-tubulin (Cat# 04-1049; WB 1:2000) was purchased from Millipore Sigma. Rabbit anti-pan-tau (Cat# A0024; WB 1:2000) was purchased from Agilent-Dako. Mouse anti-phospho-tau pThr181 (Cat# MN1050; WB 1:500), rabbit anti-phospho-tau pSer202/Thr205 (AT8) (Cat# MN1020; IF 1:200 and WB 1:500), rabbit anti-phospho-tau pSer214 (Cat# 44-742G; IF 1:50 and WB 1:500), rabbit anti-phospho-tau pThr231 (Cat# 701056; WB 1:500), rabbit anti-phospho-tau pSer262 (Cat# 44-750G; WB 1:500), rabbit anti-phospho-tau pSer199/Ser202 (Cat# 44-768G; WB 1:500) antibodies were purchased from ThermoFisher Scientific. Human/murine phospho-tau pSer396/ Ser404 (PHF1; WB 1:1000) monoclonal antibodies was provided by Peter Davies. Chicken anti-MAP2 (Cat# ab92434; IF 1:500) was purchased from Abcam. Mouse anti-APP (Cat# SIG-39320; WB 1:2500) was purchased from BioLegend. Anti-Rabbit HRP (Cat# R1006; WB 1:1000) and anti-Mouse HRP (Cat# R1005; WB 1:1000) were purchased from Kindle Biosciences. Alexa Fluor 568 goat anti-rabbit IgG (Cat# A11011; IF 1:1000), Alexa Fluor 488 goat anti-mouse IgG (Cat# A11001; IF 1:1000) and Alexa Fluor 647 goat anti-chicken IgG (Cat# A21449; IF 1:1000) secondary antibodies were purchased from ThermoFisher Scientific.

### Western blotting

10 μg of total protein was resolved by sodium dodecyl sulphate-polyacrylamide gel electrophoresis (SDS-PAGE) and transblotted using standard procedures. Nitrocellulose membranes (BioRad) were blocked in TBS-T (150 mM NaCl, 20 mM Tris–HCl, pH 7.5, 0.1% Tween 20) supplemented with 5% non-fat dry milk. Membranes were incubated overnight at 4 °C with designated primary antibodies (see above) in TBS-T supplemented with 5% non-fat dry milk, washed with TBS and next incubated with HRP-conjugated secondary antibodies (Kindle Biosciences; Cell Signaling Technology) for 1 h. Afterwards, the membrane was washed with TBS-T and developed using the chemiluminescence ECL kit (Millipore Classico or Kindle Biosciences ECL kit) and imaged using a Kwik Quant Imager (Kindle Biosciences).

For Western blotting of Aβ oligomers, 10–20% Tris–Tricine SDS-PAGE was performed. Synthetic AβO were prepared in 1X Laemmli sample buffer (Bio-Rad Laboratories) without reducing agent and resolved by SDS-PAGE. The separated proteins were transferred onto Immobilon-FL PVDF membrane (EMD Millipore), and subsequently blocked and incubated with 6E10 monoclonal antibody and secondary antibodies (see Supplementary Table 2) as indicated above for nitrocellulose membranes.

### Cell viability assay

Neuronal cultures were treated with AβO preparations and cell viability was determined using the CellTiter-Glo Luminescent Cell Viability Assay (Promega) following the manufacturer’s instructions. Neurons were infected with NSun2 expression virus 5 days prior to AβO treatment.

### RNA isolation

Fresh-frozen pulverized brain tissues or rat primary hippocampal neurons were lysed in QIAzol and homogenized using a QIAshredder column. Total RNA was extracted using the miRNeasy Kit (Qiagen). RNA concentration and purity were assessed by measuring the optical density at 260 and 280 nm with a Nanodrop Spectrophotometer (ThermoFisher).

### RNA immunoprecipitation

For immunoprecipitation of RNA, two rounds using 5 μg of anti-m^6^A antibody and 4 μg of small RNA were performed. The reaction was carried out using the Immunoprecipitation Kit-Dynabeads Protein G (ThermoFisher) following previously established protocols with slight modifications [62]. First, the anti-m^6^A antibody (see above) was coupled to Dynabeads Protein G in 500 μl of Binding and Washing Solution for 3 h at 4 °C followed by 10 min incubation at room temperature. Beads were then washed three times in Washing Buffer. Small RNA was added to the antibody-coupled beads in 1X IP buffer (10 mM Tris–HCl, 150 mM NaCl and 0.1% (vol/vol) Igepal CA-630) supplemented with Rnase inhibitor-RNAsin Plus (Promega) and kept on the rotating platform at 4 °C overnight. On the next morning, beads were washed 4 times with IP buffer. Finally, the beads were resuspended in 100 μl of m^6^A competitive elution buffer (1X IP buffer containing 6.7 mM *N*^6^-methyladenosine 5′-monophosphate sodium salt (Sigma-Aldrich) and RNAsin Plus) with continuous shaking for 1 h at 4 °C. The mixture was placed on a magnetic separation rack, and supernatant containing the eluted m^6^A RNA was collected to a new tube. Then, another 100 μl of m^6^A competitive elution buffer was added for one more elution. Immunoprecipitated RNA was recovered using the miRNeasy Kit (Qiagen) following the instructions from the manufacturer. As a control, immunoprecipitation was performed using IgG instead of anti-m^6^A antibody. The rest of experimental parameters were kept identical.

### Quantitative real‐time PCR

*c*DNA synthesis was performed using the First Strand cDNA Synthesis Kit (Origene) following the manufacturer’s instructions. Quantitative real-time PCR (QPCR) was performed using *Power* SYBR Green PCR Master Mix (Applied Biosystems) and an Eppendorf Realplex Mastercyler with the following settings: 1 cycle at 95 °C for 10 min and 45 cycles of amplification: 95 °C for 30 s, 57 °C for 1 min and 60 °C for 30 s. The following primers pairs were used: human NSun2 Fwd 5′-ATGATCGAATTTTATGTGATGTCC-3′ and human NSun2 Rvrs 5′-AGGTGGTCCACTTTTTCCAA-3′; human GAPDH 5′-CTGCACCACCAACTGCTTAG-3′ and human GAPDH Rvrs 5’-GTCTTCTGGGTGGCAGTGAT-3′; rat NSun2 Fwd 5′-GTCAGCGAGACGGAGTCTG -3′ and rat NSun2 Rvrs 5′-TGGGGGAATCATGCTGAC-3′; rat GAPDH 5′-GGCAAGTTCAATGGCACAGT-3′ and rat GAPDH Rvrs 5′-TGGTGAAGACGCCAGTAGACTC-3′. The amount of NSun2 was quantified and normalized to GAPDH mRNA using the comparative CT method.

TaqMan MicroRNA assays were used to measure miR-125b-5p levels (ThermoFisher). 10 ng of total RNA was reverse-transcribed using specific stem-loop reverse transcription primers (ThermoFisher) and miR-125b-5p levels were measured on an Eppendorf Realplex Mastercyler. The levels of U6 snRNA were used as endogenous controls for normalization using the comparative CT method.

### Immunofluorescence

For immunofluorescence of human brain sections, human FFPE sections were deparaffinized with Histoclear and rehydrated before performing antigen retrieval. Antigen retrieval procedure was done using Citrate buffer (Biogenex). FFPE sections were blocked in TBST containing 10% normal goat serum for 1 h at room temperature and incubated with primary antibody (see above) in TBST containing 10% normal donkey serum at 4 °C overnight. After three washes with TBST, the tissues were incubated with Alexa Fluor 488 goat anti-rabbit or Alexa Fluor 594 goat anti-mouse IgG (see above) for 3 h at room temperature. Following three washes with TBS, autofluorescence was quenched with 0.3% Sudan black B (Sigma-Aldrich) in 70% ethanol (Fisher Scientific) for 20 min at room temperature. The sections were rinsed in TBS. The nuclei were stained Hoechst33342 staining dye solution (ThermoFisher) in TBS for 8 min at room temperature. Following three washes with TBS, sections were mounted on slides using SlowFade gold anti-fade reagent (ThermoFisher) and imaged using confocal laser-scanning microscopy via Z stack.

For immunofluorescence of human iPSC-derived neurons, we follow previously established methods [26]. Briefly, iPSC-derived neurons on coverslips were fixed with 4% paraformaldehyde (Electron microscopy) and permeabilized with 0.2% in TBS. Coverslips were quenched with ammonium chloride for 10 min then blocked with 10% normal goat serum for 30 min. Next, coverslips were incubated with primary antibodies for NSun2, phospho-Ser214 tau and MAP2 (see above). Anti-mouse, anti-rabbit, and anti-chicken secondary antisera conjugated with Alexa Fluor dyes (ThermoFisher-Life Technologies) were used for secondary immunodetection (see above). Finally, coverslips were stained with DAPI for 10 min and mounted using Shandon Immu-Mount mounting medium (Fisher Scientific). Labeled neurons were imaged using confocal laser-scanning microscopy via Z stack.

For immunofluorescence of rat primary neuronal cultures, cells on coverslips were fixed with 4.0% paraformaldehyde (Electron microscopy) and blocked with 10% normal goat serum in TBS (ThermoFisher) and incubated overnight in NSun2 antisera (ThermoFisher), AT8 antisera (ThermoFisher) and MAP2 antisera (Abcam). Anti-rabbit, anti-mouse and anti-chicken secondary antisera conjugated with Alexa Fluor dyes (ThermoFisher-Life Technologies) were used for immunodetection. Labeled neurons were imaged in a Zeiss confocal laser-scanning microscope (LSM800).

### Single cell data analyses

The raw datasets for human superior frontal gyrus (SFG) and entorhinal cortex (EC) samples were downloaded from the Gene Expression Omnibus (GEO) repository under the following GEO ID: GSE147528 [52]. Cell filtering, cell clustering and main cell-type identification was performed as described [21]. Genes that show differential expression in at least two cell clusters of the neurons are selected for preparing the graphs. Difference to no-change baseline is calculated by non-parametric test with uncorrected Dunn’s test.

### Differential gene expression analyses

To identify differentially expressed genes, we compared the main cell types, using Braak stage 6 compared to Braak stages 0 or 2 in EC and SFG samples using FindMarker in Seurat with default options except of the followings; logfc.threshold = 0.1, min.pct = 0.0 and min.diff.pct = 0.0. We checked the expression of genes documented in this study. *p < 0.0332; **p < 0.0021; ***p < 0.0002, ****p < 0.0001.

### Public mass spectrometry dataset analyses

To check the expression of genes in mass spectrometry datasets, we downloaded the raw expression values from publicly available datasets and curations [5, 89] with a custom R script (available upon request). The protein expression and their fold change in each tissue are calculated on raw peptide reads. Mean values of different analyses on 7 platforms/datasets are shown as raw values and means.

### Statistical analysis

For quantitative immunoblotting, rough eye phenotype assessment and for Western blot and QPCR experiments, the statistical significance was determined by Student’s t test or Mann–Whitney *U* test using GraphPad Prism (GraphPad Software, La Jolla, CA, USA). One-way ANOVA with Bonferroni post hoc test was applied for comparisons including more than two groups. A *p* value of less than 0.05 was considered significant. For all figures in which error bars are shown, data represent the mean ± SEM. Statistical outliers and specimens with measurement errors were excluded.

### Study approval

The studies using de-identified post mortem autopsy tissue were reviewed and approved by the Columbia University institutional review board (IRB) (New York, NY). *Drosophila* studies are not subject to IRB oversight.

## Results

### NSun2 RNA methyltransferase is dysregulated in Alzheimer’s disease

**Fig. 1:**
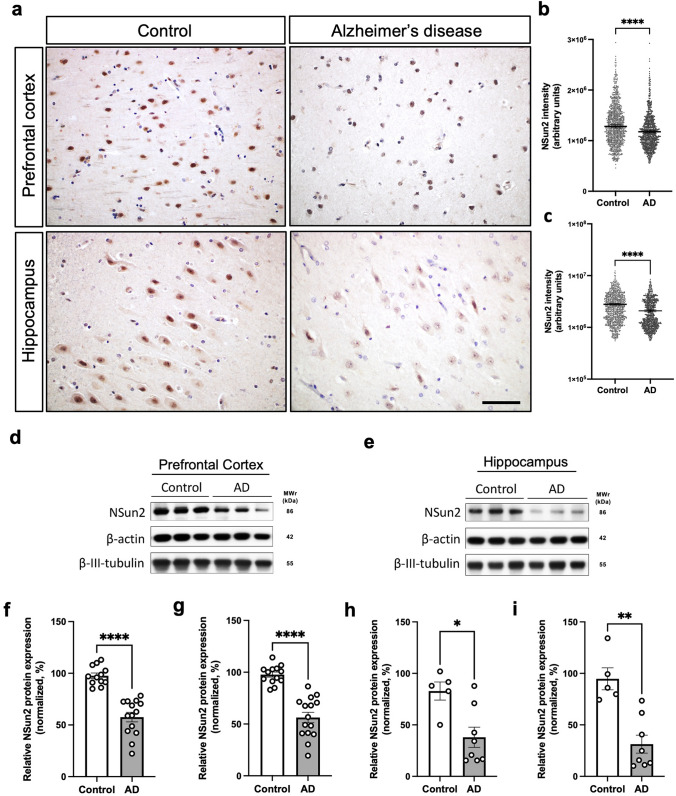
NSun2 RNA methyltransferase is downregulated in Alzheimer’s disease. **a** Representative NSun2 immunohistochemistry images of the hippocampal formation (CA1) and prefrontal cortex (Brodmann area 9) of controls and Alzheimer’s disease (AD) human brains. Quantifications of the intensity of NSuns2 in cells within the prefrontal cortex (**b**) and CA1 (**c**). Western blots for NSun2, β-actin and β-III-tubulin on the prefrontal cortex (**d**, **f**, **g**) and hippocampus (**e**, **h**, **i**) with densitometry quantification of NSun2 protein normalized to β-actin (**f**, **h**) and β-III-tubulin (**g**, **i**). Scale bar in a = 50 μm. *p < 0.05, **p < 0.01, ****p < 0.0001 by Mann–Whitney *U* test. Data represent mean ± SEM

To provide evidence that NSun2 is expressed in adult human neurons in the hippocampal formation and prefrontal cortex, main brain areas affected in AD, we performed NSun2 immunohistochemistry. Immunostaining of human control brains shows NSun2 positive neurons in the hippocampal formation and the prefrontal cortex (Brodmann area 9). Immunolabeling clearly shows that NSun2 protein is expressed in neurons in the adult human brain with strong positivity in the nucleus (Supplementary Fig. 1, online resource). To determine whether alterations in NSun2 levels are a feature of AD, we performed NSun2 immunohistochemistry on brain sections from AD patients and control individuals (Fig. 1; Table 1; Supplementary Table 1, online resource). The most salient feature was the neuronal nuclear decrease of NSun2 immunoreactivity in both brain regions of AD patients compared to controls, as confirmed by quantification of immunostained slides (Fig. 1a–c).

We then performed Western blot analysis to confirm whether levels of NSun2 are reduced in AD brains. Immunoblots using antisera specifically recognizing human NSun2 [37] (Supplementary Fig. 1, online resource) show a decrease in the levels of NSun2 in both the prefrontal cortex (Fig. 1d, f, g) and hippocampal formation (Fig. 1e, h, i), in comparison to samples from control subjects. The reduction was more pronounced in the hippocampal formation than in the prefrontal cortex (54.1% versus 41%) (Fig. 1d–i). Importantly, no significant difference was observed when comparing AD cases and controls in the cerebellum, a brain area devoid of AD pathology (Supplementary Fig. 2, online resource). Notably, we did not detect a difference in the beta-III-tubulin neuronal marker [13] indicating that our observed differences might not be secondary to neuronal loss (Fig. 1d, g, e, i). However, while quantitative real-time PCR (QPCR) analysis did not show a significant difference in the levels of NSun2 mRNA in the hippocampal formation, prefrontal cortex NSun2 mRNA levels were significantly higher in AD brains, suggesting a compensatory mechanism in the prefrontal cortex, a brain area that degenerates later in the disease process (Supplementary Fig. 3, online resource). To further explore NSun2 expression in AD patient brains, we analyzed publicly available independent single cell transcriptomics and proteomics datasets for m6A regulatory proteins including ‘writers’ (methyltransferases), ‘erasers’ (demethylases) and ‘readers’ in human AD and control brains [89, 94] (Supplementary Fig. 4, online resource). This analysis confirmed and validated our observations of NSun2 downregulation in AD patient brains.

**Fig. 2:**
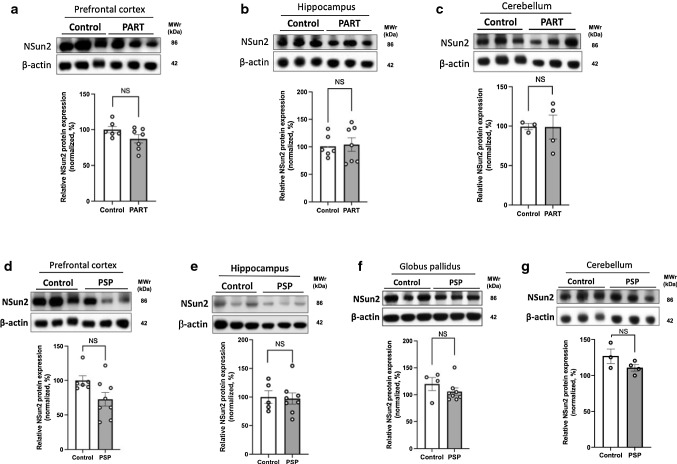
NSun2 protein levels in human primary tauopathies. Western blots for NSun2 and β-actin were performed on postmortem brain tissue from patients with Primary Age-Related Tauopathy (PART) with dementia (**a**–**c**), Progressive Supranuclear Palsy (PSP) (**d**–**g**) and control individuals (**a**–**g**). The Western blots for NSun2 and β-actin were performed on the prefrontal cortex (**a**, **d**), hippocampus (**b**, **e**), cerebellum (**c**, **g**) and globus pallidus (**f**). Densitometry quantification of NSun2 protein normalized to β-actin did not reveal significant differences in the levels of NSun2 protein between control, PART and PSP groups. Mann–Whitney *U* test was applied. Data represent mean ± SEM

To examine whether downregulation of NSun2 is a common event among other tauopathies we performed NSun2 Western blot analysis using brain samples from Primary Age-Related Tauopathy (PART) with dementia [22], Progressive Supranuclear Palsy (PSP) [30, 81] and control cases (Table 1; Supplementary Table 1, online resource; Fig. 2). We did not observe any decrease in the levels of NSun2 in the tissues from the prefrontal cortex, hippocampal formation and cerebellum (PART versus controls; PSP versus controls) (Fig. 2a, b, d, e, c, g) and from the globus pallidus (PSP versus controls) (Fig. 2f), suggesting that the reduction of NSun2 is a selective feature of AD brains when compared to other tauopathies.

**Fig. 3:**
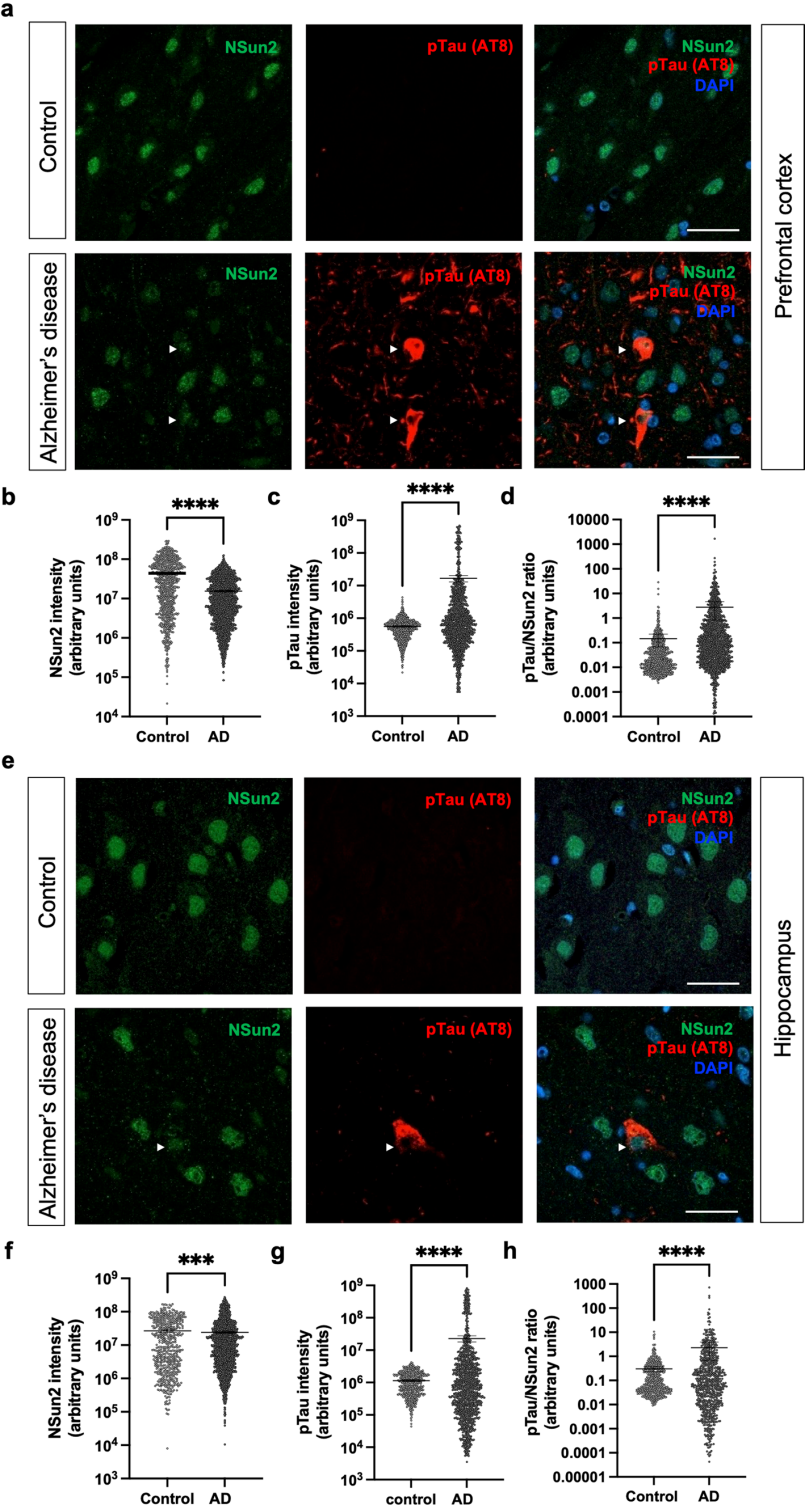
Neuronal NSun2 deficiency is associated with increased phospho-tau expression in Alzheimer’s disease brains. Representative images of immunostainings for AT8 (red) and NSun2 (green) of **a** the prefrontal cortex and **e** the hippocampus (CA1) in control individuals and patients with AD with quantifications of the intensity of NSuns2 (**b**, **f**) and AT8 (**c**, **g**) and their ratio (**d**, **h**) in cells within the prefrontal cortex (**b**–**d**) and CA1 (**f**–**h**). Note that the y-axis is presented as log10 scale. Scale bars in **a** and **e** = 50 μm. ***p < 0.001, ****p < 0.0001 by Mann–Whitney *U* test. Data represent mean ± SEM

Since AD patients show pronounced tau pathology in the prefrontal cortex and hippocampus, we wondered if reduced expression of NSun2 in these areas was paralleled by an increase in phospho-tau expression within the same neurons. To address this question, we performed co-immunostainings for phospho-tau (AT8 immunostaining) and NSun2 in human AD brain sections of the prefrontal cortex (Fig. 3a–d) and hippocampus (Fig. 3e–h). Quantifications of these stainings revealed significantly higher levels of phospho-tau (prefrontal cortex: 2957%; hippocampus: 2063%) and significantly reduced levels of NSun2 (prefrontal cortex: − 64.8%; hippocampus: − 9.1%) in neurons of the AD group (Fig. 3b, c, f, g) with an increased phospho-tau/NSun2 ratio at a cellular level (1806% in frontal cortex, 653% in hippocampus) (Fig. 3d, h).

### Downregulation of NSun2 in human neurons results in increased tau phosphorylation

**Fig. 4:**
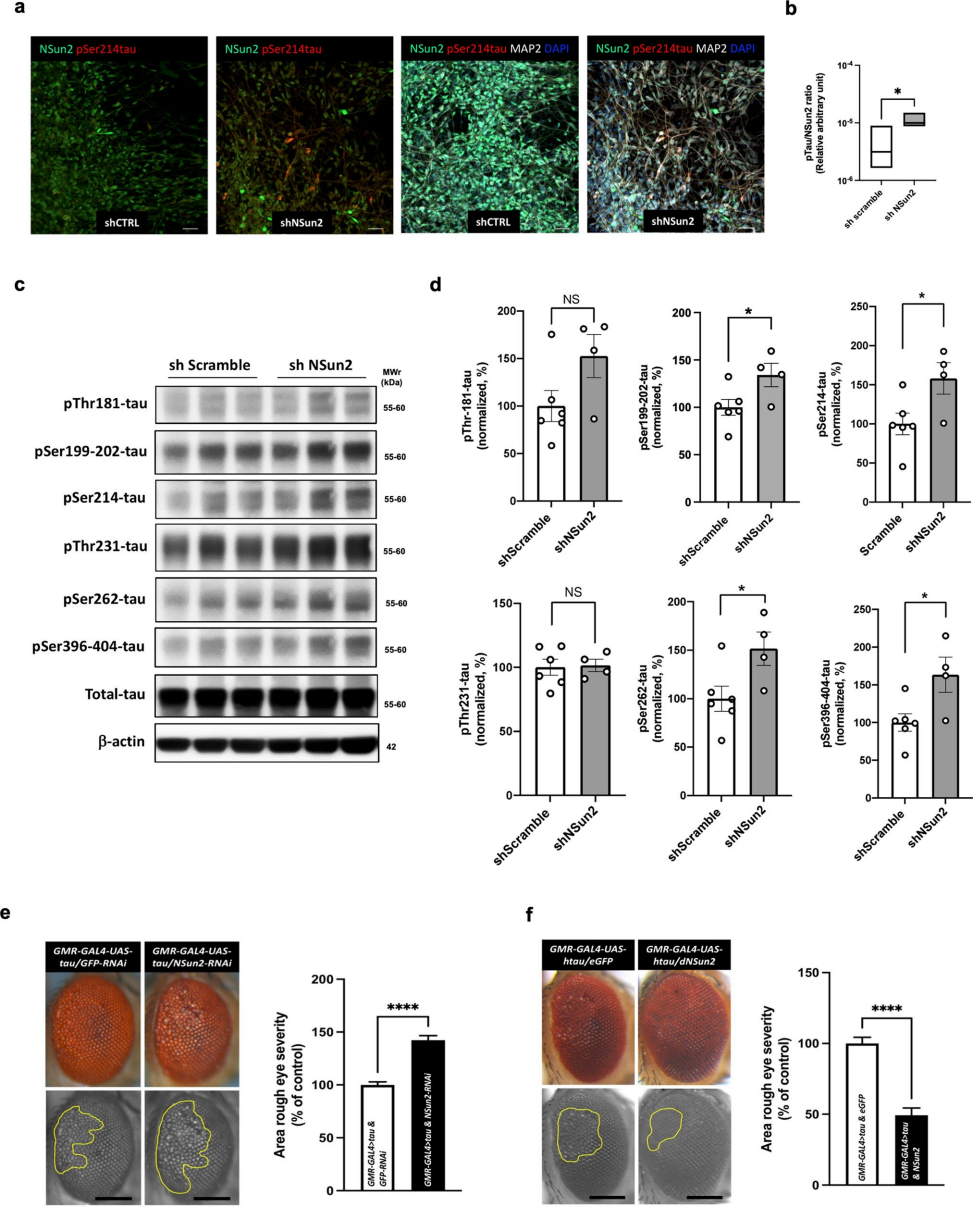
NSun2 deficiency promotes tau hyperphosphorylation and tau toxicity. **a** Representative immunofluorescence images of human iPSC-derived neurons transduced with shNSun2 or scramble control immunostained using NSun2 (green), phospho-serine 214-tau (pSer-214-tau; red) and MAP2 (white) antibodies. **b** Quantification of the intensity of NSun2 and pSer214-tau expression in shNSun2 or scramble control neurons presented as ratio. **c** Representative Western blots with indicated antibodies demonstrating the effects of shNSun2 on the levels of phosphorylated forms of tau. **d** Quantification of phosphorylated tau in neurons transduced with shNSun2, plotted relative to the levels of total tau (after normalization of total tau with β-actin levels). *p < 0.05 by Student’s *t* test. Data represent mean ± SEM. **e**, **f** Regulation of tau toxicity by NSun2 in drosophila tau models. **e** Co-expression of NSun2-RNAi (*n* = 26) with human tau exacerbated the rough eye phenotype compared with that observed in the GFP-RNAi control (*n* = 29). **f** Co-expression of NSun2 (*n* = 18) partially suppressed the human tau–induced rough eye phenotype compared with that seen in the GFP control (*n* = 28). The yellow marked area shows the degenerated part of eyes. Scale bars, 200 μm. Histograms show quantitative assessment of eye phenotypes. ****p ≤ 0.0001 by Mann–Whitney *U* test. Data represent mean ± SEM

Having observed that NSun2 is reduced in human samples and that NSun2 deficient neurons present higher levels of phospho-tau in human AD brains, we next asked whether reduction of NSun2 might trigger tau proteostasis dysregulation by altering tau phosphorylation levels in human neurons. To this end, we took advantage of iPSC-derived neurons as a favored in vitro model system to interrogate and investigate molecular events driving tau dysregulation in humans [43, 65, 70]. Efficient gene knockdown was tested using a pool of short hairpin RNA and its respective scramble control. Our system enabled us to significantly reduce NSun2 protein levels (31.88% decrease, *p* = 0.0018) in the iPSC-derived neuronal cultures (Supplementary Fig. 5, online resource), resembling a similar reduction observed in the human cortex of AD brains. We then performed immunostainings on the human iPSC-derived neuronal cultures and found an increase in the number of positive phospho-serine-214-tau neuronal cells and an increase in the phospho-tau/NSun2 ratio upon NSun2 protein knockdown compared to controls (Fig. 4a, b). Strikingly, Western blot analysis of whole cell lysates using a battery of anti-phospho-tau antibodies (Fig. 4c) showed that NSun2 knockdown promoted a significant increase in the levels of phosphorylated tau in several of the phospho-epitopes tested (pSer-199-202, pSer-214, pSer-262, pSer-396-404) (Fig. 4d).

Similar effects were also observed when we reduced the expression of NSun2 in rat primary hippocampal neurons from wild-type rats. Here, shRNA mediated knockdown of NSun2 protein resulted in significantly increased expression of tau pSer-214 and tau pThr-231 (Supplementary Fig. 6, online resource).

### Deleterious effect of NSun2 deficiency in a *Drosophila* melanogaster model of tau toxicity

We then investigated whether NSun2 influences tau toxicity in vivo. Many molecular mechanisms of post-transcriptional regulation including epitranscriptomic modifications and miRNA regulation are conserved between *Drosophila* melanogaster (fruit flies) and humans [23]. Furthermore, fruit flies have proven to be useful for modeling essential mechanisms of tauopathy and tau biology [67]. Using a conditional expression system, we overexpressed either a short interfering RNA (siRNA) against NSun2 or we overexpressed NSun2. First, human tau was co-expressed with a siRNA control in the *Drosophila* eye, producing a rough eye phenotype, confirming that the model system was functioning (Fig. 4e). Next, human tau and NSun2 siRNA were co-expressed resulting in an exacerbation of the phenotype (Fig. 4e). In contrast, co-expression of human tau and *Drosophila* NSun2 showed a partial reversal of the rough eye phenotype, consistent with a protective role, demonstrating bidirectionality (Fig. 4f). Quantitative assessment of these phenotypes revealed that these findings are highly significant (Fig. 4e, f), demonstrating that NSun2 influences tau toxicity in this system.

### Decreased levels of neuroprotective NSun2 promotes epitranscriptomic alterations in miR-125b and tau hyperphosphorylation

**Fig. 5:**
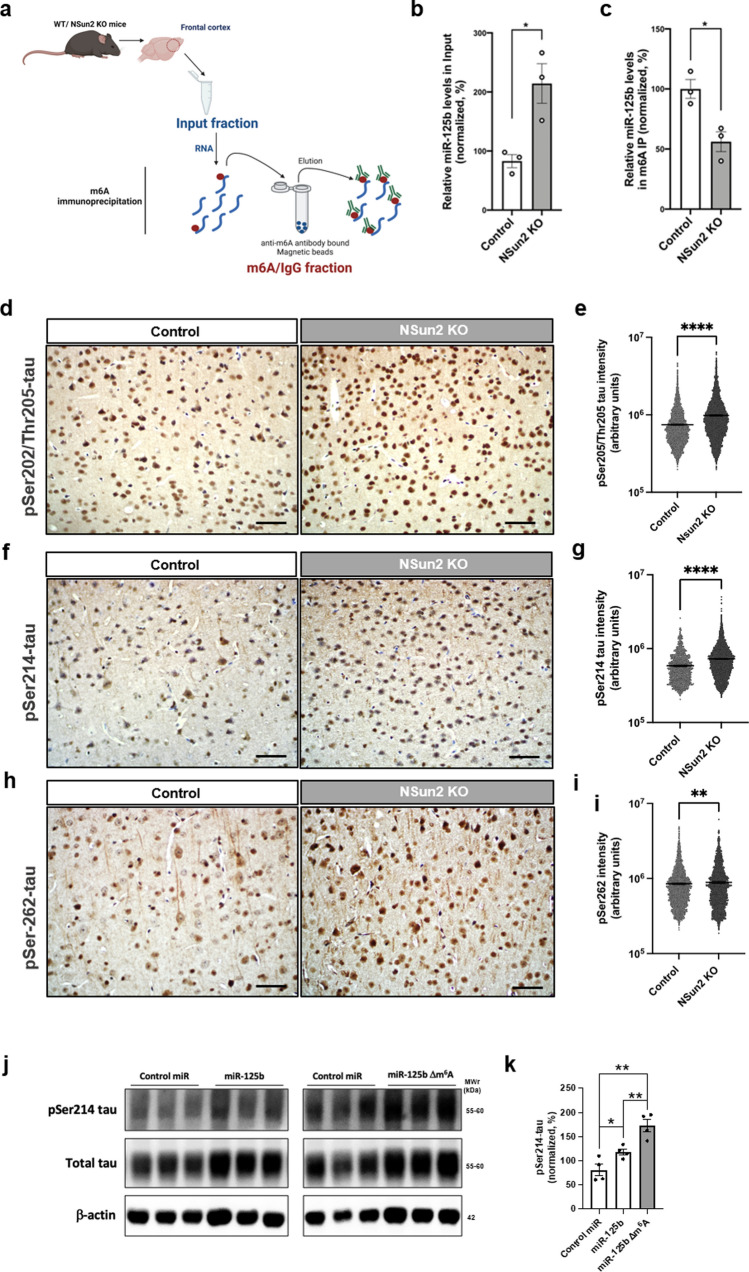
NSun2 deficiency promotes epitranscriptomic alterations in miR-125b. **a** Diagram showing that RNA was isolated from the forebrain of NSun2 conditional KO mice and non-transgenic controls and was subjected to immunoprecipitation analysis using an anti-m^6^A antibody or IgG control. **b**, **c** The presence of miR-125b in the Input (*n* = 3) m^6^A (*n* = 3) and IgG (*n* = 3; used as negative control) materials was analyzed by QPCR. Histograms show quantification of miR-125b levels with respect to control in Input (b) and m^6^A (c) materials. *p < 0.05 by Student’s *t* test. Data represent mean ± SEM. Representative images (**d**, **f**, **h**) and quantifications (**e**, **g**, **i**) of immunohistochemistry on the frontal cortex of NSun2 conditional KO and non-transgenic control mice with phospho-Serine 202-phospho-Threonine 205 (AT8) (**d**, **e**), phospho-Serine 214-tau (**f**, **g**) and phospho-Serine 262-tau (**h**, **i**) antibodies showing a marked increase in phospho-tau immunostaining in NSun2 conditional KO animals. Note that the y-axis is presented as log10 scale. Scale bars, 100 μm. **j**, **k** After site directed mutagenesis, transduction of human iPSC-derived neurons with miR-125b vector induced a significant increase of pSer-214-tau levels when compared to scrambled miR control, while cells transduced with 125b mutant vector (miR-125b∆m^6^A) exhibited significant higher levels of pSer-214-tau. *p < 0.05 and **p < 0.01 by one-way ANOVA with Bonferroni post hoc test

It has been shown that NSun2 mediates m^6^A methylation of miR-125b, repressing its processing and function [93]. Importantly, miR-125b is found to be upregulated in AD [20, 58, 78, 86] and its upregulation promotes tau hyperphosphorylation and cognitive deficits in vivo [6]. To study whether NSun2 deficiency alters m^6^A methylation of miR-125b and promotes tau hyperphosphorylation in vivo in the brain, we performed m6A-methylated RNA immunoprecipitation followed by QPCR analysis and phospho-tau immunohistochemistry (Fig. 5). RNA isolated from NSun2 KO mice brain cortical samples was immunoprecipitated using an anti-m^6^A antibody and the presence of methylated miR-125b in the immunoprecipitated materials was analyzed by QPCR (Fig. 5a). As expected, miR-125b levels were significantly increased in the input samples from NSun2 KO mice brains (Fig. 5b). Conversely, the levels of methylated miR-125b were found significantly decreased in the brain samples from NSun2-deficient mice (43.90%, *p* = 0.0182) (Fig. 5c). To further confirm NSun2 deficiency promoted tau alterations in vivo*,* we performed AT8 (pSer-202/Thr205-tau) (Fig. 5d, e), pSer-214-tau (Fig. 5f, g) and pSer-262-tau (Fig. 5h, i) immunostaining on the brains of aged NSun2 conditional knockout animals. Strikingly, immunoreactivity for AT8, pSer214-tau and pSer-262-tau was significantly increased in neurons in the frontal cortex of NSun2 deficient mice (31.8%, 23.9% and 4.4%, respectively) (Fig. 5e, g, i).

Next, we wanted to confirm in our AD brain tissues samples whether miR-125b is found upregulated. We found that the levels of miR-125b were significantly increased in AD frontal cortex samples which is in agreement with previously reported observations and our data on the NSun2 deficient mouse brains (Supplementary Fig. 7, online resource).

To further investigate the potential role of miR-125b m6A methylation on tau phosphorylation, we substituted the m6A motif site AAC with GGC in the lentiviral vector encoding miR-125b (miRNA sequence UCCCUGAGACCCUAACUUGUGA). Transduction of iPSC-derived neurons with miR-125b vector induced a significant increase of pSer-214-tau levels when compared to scrambled miR control (Fig. 5j, k) which confirms similar previously reported results in rodent neurons [6]. Strikingly, cells transduced with 125b mutant vector (miR-125b∆m6A) exhibited significantly higher levels of pSer-214-tau (Fig. 5j, k), supporting the role of m6A miRNA methylation in regulating tau phosphorylation.

### Oligomeric amyloid-beta induces neuronal loss of NSun2

**Fig. 6:**
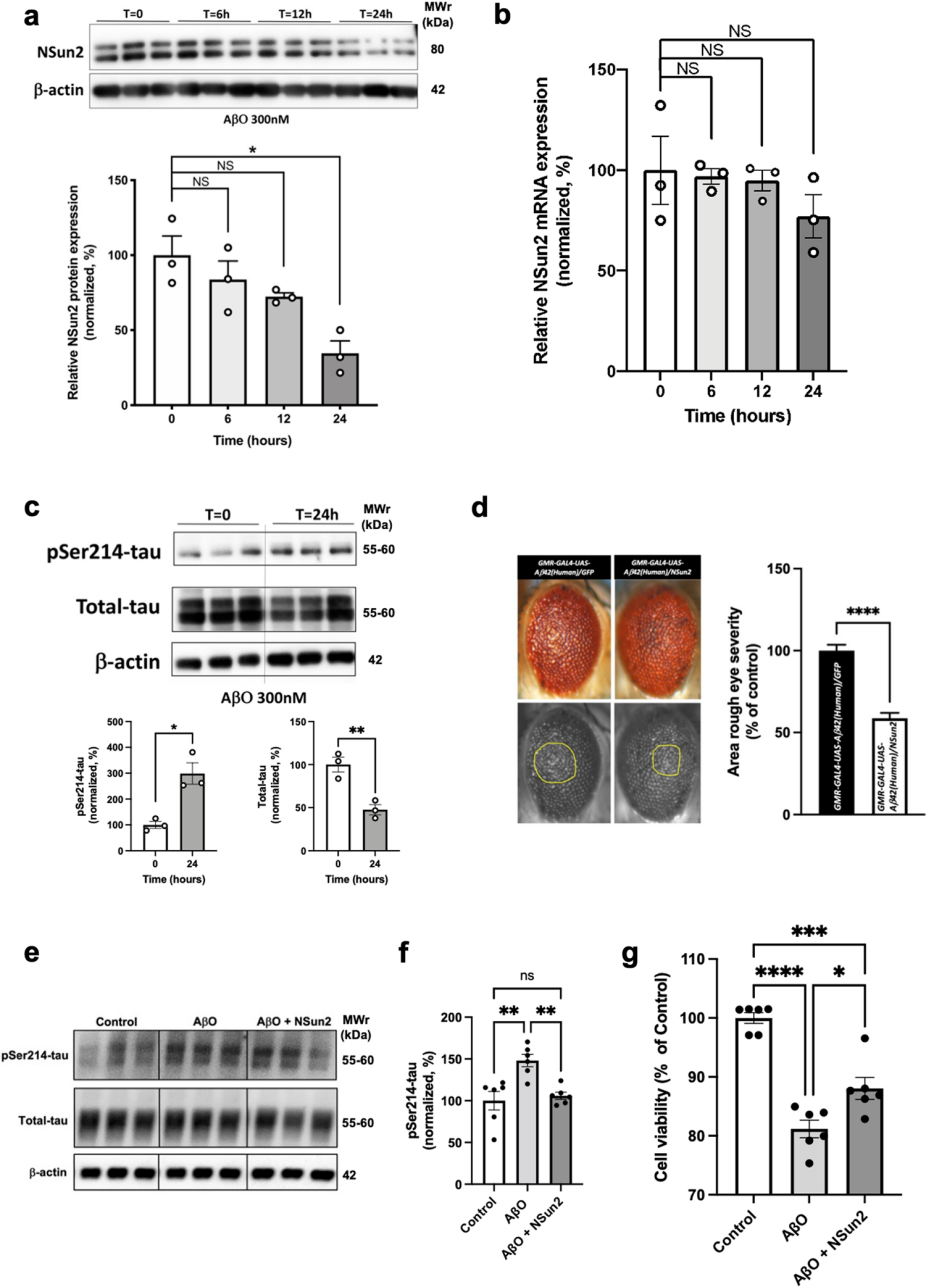
NSun2 is downregulated in response to amyloid-β oligomers. **a** Western blot quantification of NSun2 protein levels in rat primary hippocampal neurons untreated (control) or treated with 300 nM AβO for the indicated times. Top portion of the panel shows representative Western blots. Histograms show densitometry quantification of NSun2 protein levels (bottom of the panel). NSun2 values are normalized against β-actin. Data represent mean ± SEM. *p < 0.05 by One-way ANOVA with Bonferroni post hoc test. **b** QPCR analysis of NSun2 mRNA levels in rat primary hippocampal neurons untreated (control) or treated with 300 nM AβO for the indicated times. No significant changes in the levels of NSun2 mRNA are found. **c** Western blot quantification of phospho-tau and total tau levels in rat primary hippocampal neurons untreated (control) or treated with 300 nM AβO for 24 h. Phospho-tau levels are plotted relative to the levels of total tau (after normalization of total tau with β-actin levels; also shown here). **d** Co-expression of NSun2 (*n* = 16) partially suppressed the Aβ42-induced rough eye phenotype compared with that seen in the GFP control (*n* = 16). The marked area shows the degenerated part of eyes. Histograms show quantitative assessment of eye phenotypes. ****p ≤ 0.0001 by Mann–Whitney *U* test. Data represent mean ± SEM. **e** Human iPSC-derived neurons were treated with AβO and protein lysates were collected 24 h later for analysis. Representative Western blots with antibodies directed against pSer214-tau and total tau demonstrate the effects of AβO treatment and rescue by NSun2 overexpression in human neurons. **f** Quantification of phospho-tau in AβO-challenged neurons in the presence of absence of NSun2 demonstrating significant reduction of tau phosphorylation after overexpression of NSun2. **g** Cell viability assay demonstrating partial rescue of cell viability in AβO-challenged neurons after overexpression of NSun2. *p < 0.05, **p < 0.01, ***p ≤ 0.001 and ****p ≤ 0.0001 One-way ANOVA with Bonferroni post hoc test. Data represent mean ± SEM

One of the main factors that distinguishes AD from primary tauopathies is the accumulation of AβO in the brain, which is positively correlated with cognitive decline and/or disease progression in AD patients and animal models [32]. Given the significant reduction of NSun2 levels in AD brains (Fig. 1), we tested whether Aβ could trigger downregulation of NSun2. To this end, rat primary hippocampal neurons from wild-type rats were exposed to 300 nM AβO, a sub-apoptotic concentration (Fig. 6a–c; Supplementary Fig. 8, online resource). We found that NSun2 protein levels decreased over 24 h AβO exposure (Fig. 6a) while NSun2 mRNA levels were not affected (Fig. 6b). Concomitantly, we observed a significant increase in phospho-tau levels using an anti pSer-214-tau antibody at 24 h paralleled by significant reduction of total tau (Fig. 6c).

### NSun2 partially rescues amyloid-beta-induced toxicity

We next investigated whether NSun2 influences Aβ-induced toxicity in vivo. Using the human Aβ42 transgenic *drosophila* model that also develops a rough eye phenotype, we overexpressed either GFP or NSun2 in these flies (Fig. 6d). Notably, NSun2 overexpression resulted in a significant reversal of the rough eye phenotype, as similarly seen after NSun2 overexpression in our human tau overexpression drosophila model. These observations demonstrated that NSun2 partially rescues Aβ-induced toxicity in vivo.

To investigate if NSun2 also rescues AβO-induced toxicity in human neurons, we next exposed human iPSC-derived neurons to AβO. This treatment caused significant upregulation of phospho-tau (pSer-214-tau), as similarly observed for rat primary neurons (Fig. 6e, f). Thus, we decided to investigate if overexpression of NSun2 would lead to reduction of phospho-tau levels in these human cells. Notably, NSun2 overexpression resulted in significantly reduced levels of phospho-tau in AβO-challenged neurons (Fig. 6e, f). In addition, overexpression of NSun2 rescued cell viability and reduced AβO-induced toxicity in cell viability assays (Fig. 6g), indicating a neuroprotective role of Nsun2.

## Discussion

NSun2 is an RNA methyltransferase that has been well characterized in higher eukaryotes [1, 11, 28, 38, 40, 74, 85]. It is a member of the NSun family and its expression is enriched in the developing brains [19]. Here, we have shown for the first time that NSun2 protein is expressed in the adult human brain (Fig. 1; Supplementary Fig. 1, online resource). We observed positive NSun2 staining mainly in neurons; yet, NSun2 mRNA is also found in the transcriptome profile of other cell types in the brain such as oligodendrocytes, microglia, astrocytes and endothelial cells [96, 97], indicating potential cell-type specific roles of NSun2 during development and in cellular stress responses [10].

To date several studies have implicated epitranscriptomic regulation of coding and non-coding RNAs in diverse biological brain functions [24, 77]. However, current understanding of the role that epitranscriptomic alterations play in brain dysfunction and pathology is limited [24, 87, 91]. Here, we show that NSun2 RNA methyltransferase protein levels are decreased in AD brains denoting epitranscriptomic alterations in the AD process (Fig. 1). NSun2 deficiency has a negative impact on learning and memory in fruit flies, mice and causes intellectual disability and neurological abnormalities in human [1, 12, 44]. Our results show that the brain area that is devoid of AD pathology such as cerebellum has comparable NSun2 levels to control brains, indicating that NSun2 deficiency is specific to the brain areas that are majorly affected in AD. Unexpectedly, our results also show that NSun2 protein is not reduced in primary tauopathies like PART and PSP (Fig. 2), indicating a distinct NSun2-related disease mechanism in the AD brains. Future studies will be important to uncover whether NSun2 alterations are occurring in specific disease stages or throughout the AD process.

By utilizing the tau toxicity *Drosophila* system (Fig. 3), we were able to show that NSun2 could modulate tau toxicity bidirectionally. A partial rescue of the rough eye phenotype was observed when NSun2 is overexpressed suggesting that NSun2 can play a protective role against tau toxicity, and this supports previously published neuroprotective roles of NSun2 [10].

Even though m^6^A methylation occurring in miRNAs has been reported in few studies before [4, 46, 93], none of these studies had investigated m^6^A miRNA methylation in the brain. NSun2 is one of the few brain-enriched methyltransferases known to facilitate methylation of non-coding RNAs, including miRNAs. It has been shown that NSun2 methylates miR-125b repressing its processing and function [93]. Moreover, miR-125b is upregulated in AD brains [20, 58, 78, 86] and its upregulation promotes tau hyperphosphorylation and cognitive deficits in vivo [6]. Here, our results on NSun2 conditional knockout mice show that NSun2 deficiency promotes alterations in miR-125b methylation and dysregulation of tau proteostasis in vivo by inducing changes in tau phosphorylation levels (Fig. 5). Notably, it has been reported that miR-125b upregulation in neurons resulted in increased tau phosphorylation at S202/T205 (AT8) sites through silencing of phosphatase PPPCA1 [6]. We found elevated levels of AT8 staining in NSun2 conditional KO mice suggesting that PPPCA1 phosphatase could be regulated by miR-125b in the NSun2 deficient mice. As for phospho-Ser214-tau, a particular phosphatase that dephosphorylates this residue has not been identified yet. However, Akt/protein kinase B (Akt) can phosphorylate tau at Ser214 [49] and this implies that Akt might be altered in NSun2 KO mice. Future studies are needed to better untangle the complexity of NSun2 targets which may include other potential miRNAs associated with AD pathogenesis such as miR-106b [57].

Even though NSun2 has been traditionally considered an m^5^C methyltransferase and tRNAs are the major substrates of this RNA methyltransferase, our data analysis shows that NSun2 mediates m^6^A methylation in miRNAs. Indeed a possible cooperative mechanism between NSun2 and Mettl3 has been proposed [54] requiring future research. In addition, loss of NSun2 results in accumulation of tRNA fragments [9]. Curiously, tRNA fragments function as short RNAs with multi-faceted roles in disease processes [61, 84], including neurological disorders and possibly including AD [34, 60, 71, 88, 95]. Therefore, further analysis will be required to uncover other salient roles of NSun2 on the post-transcriptional regulation of other non-coding small RNAs in AD and related neurological disorders.

Remarkably, we were able to demonstrate that phospho-tau levels were upregulated upon NSun2 knockdown confirming our hypothesis that NSun2 modulates tau proteostasis mediating changes in tau phosphorylation in human neuronal cultures (Fig. 4). The human iPSC-derived neuronal culture used in this study was obtained following a well-established differentiation protocol in the laboratory that produces a heterogeneous population of neurons including glutamatergic, cholinergic neurons, GABAergic neurons and dopaminergic neurons [26]. This led us ponder if there could be a differential vulnerability to changes in NSun2 levels depending on the neuronal cell types. Even though NSun2 seems to be expressed in different neuronal cell types at least at the transcriptomic level [8], it would be very valuable to find out in future studies whether any differential expression levels of NSun2 exist depending on the neuronal cell types produced in these cultures and whether tau hyperphosphorylation induced by loss of NSun2 drives neurotoxicity in specific neuronal populations.

Our data indicate that reduction of NSun2 occurs in response to amyloid-beta accumulation (Fig. 6) leading to alterations in tau proteostasis. We also demonstrate rescue of Aβ-induced cell stress by NSun2 overexpression, providing tentative evidence that targeting of NSun2 could be of therapeutic value. Protease-activated receptor 2 (PAR2) modulates NSun2 and miR-125b methylation [90]. Moreover, PAR2 expression is reduced in vivo in response to amyloid-beta. Furthermore, PAR2 receptor levels are reduced in human AD brains [2], which could potentially explain our observed alterations in NSun2. Therefore, one plausible way of modulating NSun2 could be mediated through PAR2, one of the proteinase-activated receptors with profound roles in the nervous system [64].

While this study has several strengths in that we have linked decreased NSun2 expression to tau pathology and neurodegeneration in various in vitro and in vivo models including mice, flies, primary cultures, human iPSC-derived neurons, and AD patient brains, we recognize that there are also some limitations. First, while we were able to analyze the effects on NSun2 expression in neurons, future studies should explore possible NSun2 dysregulation and any effects on cellular pathology in glial cells in the context of AD and other tauopathies. In fact, we have noticed some differences when comparing the extent of consistent NSun2 downregulation in AD tissue in bulk preparations versus single cell neuron analyses, especially in the hippocampus, further encouraging future studies on NSun2 expression in non-neuronal cells in AD. In addition, a detailed in vivo analysis of NSun2 dysregulation and tau pathology in various neuronal subtypes, including glutamatergic neurons versus interneurons, and possible brain region-specific alterations in NSun2 and tau pathology, for instance in different compartments of the hippocampal formation or different cortical layers across the frontal, temporal, parietal and occipital lobes would be particularly informative. Second, here we have uncovered NSun2 alterations in AD, which we have also observed by proteomics and single cell analyses in AD brain tissue (Supplementary Fig. 4, online resource). However, deeper definition of the epitranscriptomic changes in other non-coding and coding RNAs as well as further characterization of alterations in other epitranscriptome regulatory proteins in AD such as Mettl3, HNRNPA2B1, YTHDF3 or YTHDF3 are needed (Supplementary Fig. 4, online resource) [33, 36, 42, 98]. Furthermore, comparative analyses with related tauopathies such as Corticobasal Degeneration, Pick’s diseases or other types of Frontotemporal Lobar Degeneration as well as unrelated neurodegenerative disorders are warranted. Third, since the *Drosophila* model used in this study is limited to the analysis on one single isoform, additional fly models and their brain function and behavior should be tested in the future, including the modulation of the NSun2 target miR-125b. Fourth, deeper characterization of miR-125b targets in vitro and in vivo could inform about the role that epitranscriptomic regulation plays in brain physiology and pathology.

In conclusion, our results suggest that Nsun2 modulates tau toxicity through epitranscriptomic regulation of tau proteostasis. This conclusion is based on the finding that NSun2 deficiency regulates tau phosphorylation in vitro and in vivo and that tau toxicity is bidirectionally regulated by NSun2 overexpression and inhibition in vivo. Our results are consistent with NSun2 influencing tau phosphorylation at the post-transcriptional level through miR-125b regulation, but that may differ depending on the experimental context. Further validation in other disease models and behavioral studies would be valuable. It is unlikely that NSun2 is the only methyltransferases with the ability to regulate miRNAs. However, the role of NSun2 is of critical interest, given the extraordinary and unique role this enzyme plays in physiology and pathology. Further studies will advance our understanding of the regulation of tau and AD pathogenesis and may guide us toward the development of novel therapeutic strategies.

## Supplementary Information

Below is the link to the electronic supplementary material.

Supplementary file1 (XLSX 15 KB)
